# Retinal involvement in sepsis: from the eye of the patient to the eye of the beholder

**DOI:** 10.1186/s13054-017-1721-2

**Published:** 2017-06-17

**Authors:** E. Christiaan Boerma

**Affiliations:** Department of Intensive Care, Medical Centre Leeuwarden, Leeuwarden, The Netherlands

**Keywords:** Sepsis, Fluorescence angiography, Retinal blood flow, Roth spot

In the history of mankind the importance of the eye has always extended beyond the organ itself. In ancient Egypt the “Eye of Horus” represented royal power and good health and reflected all six senses. In Greek mythology the symbolic value of the eye was already supported by detailed medical knowledge [1]. Not only symbolizing the giant one-eyed cyclops’ limited intelligence in contrast to the vigilance of Panoptes, whose body was covered by eye’s, the stories also reflected the understanding of the concepts of visual fields and acuity. In modern ophthalmology the idea of ocular involvement in systemic diseases, such as lupus and diabetes, is well established. In addition, the eye is also known as an organ that “mirrors” diseases in a distant primary organ of interest; Roth spots in endocarditis is the classic example [2].

In a recently published paper in *Critical Care* Erikson and co-workers extended these ideas to the context of sepsis [3]. They added direct observation of the retina to the already many-headed sepsis Hydra, which affects so many organs. Retinal fluorescein angiography was performed twice during the first 5 days in the intensive care unit in 31 patients with sepsis. In addition ocular pressure was determined. By doing so the authors demonstrated the feasibility of this technique in the local ICU setting, despite practical limitations. And the results were alarming. More than half of the patients displayed signs of retinal pathology, including fluorescein-leaking retinal micro-aneurysms, vitreous hemorrhages, and other retinal hemorrhages. These findings were bilateral in 75% of the affected cases. Ocular hypertension was present in 16% of all septic patients.

Such abnormalities were increasingly present in the subgroup of septic patients with a slowdown of arterial retinal blood flow, expressed as a prolonged retinal arterial filling time >8.3 s. In general these patients were sicker and had lower cardiac output. This led the authors to believe that retinal fluorescein angiography has potential to detect and characterize sepsis in a non-invasive way (with the exception of an intravenous fluorescent dye, that is). But as tempting as it may seem, such a search for the holy grail of an easily accessible organ that “represents” (all) other organs carries the risk of oversimplification. Recent examples of tonometry as a “canary of the body” and the sublingual microcirculation as a window for other organs remind us to be cautious [4, 5], especially in a disease state that is characterized by heterogeneity of blood flow within and between organs [6]. In addition, the idea to use retinal microvascular blood flow as a surrogate for intracranial perfusion is equally tempting. Previous observations have suggested that both retinal and conjunctival blood flow may, to some extent, represent cerebral blood flow changes during carotid endarterectomy [7, 8]. Others observed that retinal blood flow may reflect intracranial hypertension [9]. However, the absence of abolishment of conjunctival blood flow during angiography-proven brain death is reason to use the eye as a window for the brain with great caution [10].

Where should we go from here? Irrespective of the issue of retinal pathology as a surrogate for the effects of sepsis in other organs, what is the relevance of the findings in themselves. If more than half of sepsis patients have retinal pathology, what are the clinical consequences? Are they related to visual outcome? And do they need follow-up? The fact that, in this study, the abnormal retinal findings had resolved in a control angiography 3–6 months after hospital discharge in surviving patients is seemingly reassuring, but these need to be confirmed in larger high-risk patient cohorts. In addition it is known that processes other than sepsis, such as hypoxia or ischemia, may also upset the normal retina–blood barrier in the retinal capillaries, thereby allowing extravascular leakage of fluorescein. This warrants further studies to establish the incidence of retinal pathology in other high-risk ICU populations. And what about post-ICU check-ups for intraocular hypertension, usually unnoticed by the patient until irreversible visual damage has occurred. With an increasing ICU survival rate even for very complicated disease states such as sepsis, the ICU community is becoming more and more aware of post-ICU sequelae, also referred to as post-intensive care syndrome (PICS) [11]. In addition to ICU-acquired weakness and cognitive impairment, even a small deterioration in visual abilities may be very relevant for daily life activities, especially in the frail elderly with a pre-existing decline in function of potentially all six senses prior to and after ICU admission [12].

We thank doctor Erikson and his colleagues for their effort to cross the (imaginary) border between the ICU and the ophthalmology department. Their innovative research has made the ICU community aware of the fact that sepsis, and potentially other critically ill disease states, may affect the eyes of ICU patients. Undoubtedly this will change the eye of the beholder as well.
